# The experience of traumatic events, psychological distress, and social support: links to COVID-19 vaccine hesitancy and trends with age in a group of older Australians

**DOI:** 10.1186/s12877-024-04902-9

**Published:** 2024-03-31

**Authors:** Maria Christou-Ergos, Kerrie E. Wiley, Julie Leask

**Affiliations:** 1 Faculty of Medicine and Health, Susan Wakil School of Nursing and Midwifery, University of Sydney, Room 135, RC Mills Building A26, Sydney, NSW Australia; 2 Faculty of Medicine and Health, School of Public Health, University of Sydney, Sydney, NSW Australia; 3 Sydney Infectious Diseases Institute, Westmead Hospital, Westmead, NSW Australia

**Keywords:** Older adults, Australian survey, Vaccine hesitancy, COVID-19 vaccination, Traumatic life events, Social support, Psychological distress

## Abstract

**Background:**

Vaccination is important to reduce disease-associated morbidity and mortality in an ageing global population. While older adults are more likely than younger adults to accept vaccines, some remain hesitant. We sought to understand how traumatic events, psychological distress and social support contribute to older adults’ intention to receive a COVID-19 vaccine and whether these experiences change with age.

**Methods:**

We analysed survey data collected as part of the Sax Institute’s 45 and Up Study in a population of Australian adults aged 60 years and over. Data were derived from the COVID Insights study; a series of supplementary surveys about how participants experienced the COVID-19 pandemic.

**Results:**

Higher intention to receive a COVID-19 vaccine was associated with greater social support (adjusted odds ratio (aOR):1.08; 95%CI:1.06–1.11; *p* <.001) while lower intention was associated with personally experiencing a serious illness, injury or assault in the last 12 months (aOR:0.79; 95% CI:0.64–0.98; *p* =.03). Social support and the experience of traumatic events increased significantly with age, while psychological distress decreased.

**Conclusions:**

There may be factors beyond disease-associated risks that play a role in vaccine acceptance with age. Older Australians on the younger end of the age spectrum may have specific needs to address their hesitancy that may be overlooked.

## Background

Globally, most people are expected to live beyond their 6th decade [1]. The size and proportion of older populations is growing in every country around the world, and by 2030, 1 in 6 people will be aged over 60 [1]. The COVID-19 pandemic has presented an increased burden of disease-associated morbidity and mortality for older, compared to younger, populations [2]. Vaccination against COVID-19 remains the best protection against severe disease and death, and programs have prioritised older adults due to their increased risk of severe disease, which increases substantially with age [3]. In general, older adults are more likely than younger adults to want to receive a COVID-19 vaccine [4]. However, some older people remain hesitant [5–7].

Many factors contribute to vaccination decisions, including; past experiences, risk perceptions (of the vaccine and disease), social processes (including norms and family support), and practical considerations (such as access and affordability) [8, 9]. A small but growing body of research has recently considered the role of decreased psychological wellbeing and the experience of traumatic life events in contributing to vaccine hesitancy [10–13]. A traumatic event is one that causes − or has the potential to cause − death, serious injury, or violence [14]. In older populations, elder abuse, falls, and life-threatening diagnoses are prevalent and have the potential to induce psychological distress [15–17]. Additionally, limited social support in the aftermath of a distressing experience is a risk factor for decreased psychological wellbeing [18].

In Australia, older adults generally perceive themselves as healthy and socially supported [19]. However, data on older Australians are often based on comparisons with younger age groups which can overlook the nuances of age-related change within the older Australian sub-group. Additionally, data on vaccine acceptance tends to look at older populations as one large group rather than considering factors that change with age. There has yet to be an investigation of how social support, psychological distress and traumatic events relate to vaccination intention in an older Australian population. This study sought to understand how these factors contribute to older Australians’ intention to receive a COVID-19 vaccine and whether these experiences change with age. Given the ageing Australian population, this information may be valuable for designing future vaccination interventions for vaccine-hesitant older populations. Over and above COVID-19 vaccination, this may guide efforts to increase acceptance of vaccines recommended for older populations in the National Immunisation Program.

## Methods

We analysed survey data collected as part of the Sax Institute’s 45 and Up Study, an Australian longitudinal study of health and ageing that commenced recruitment in 2005 and by 2009, recruited a cohort of 267,357 Australians aged 45 and over. This study is described in detail elsewhere [20]. Participants of the 45 and Up Study were randomly sampled from the Services Australia Medicare enrolment database, which provides near complete coverage of the population. People aged 80 years and over, and those who live in rural or remote areas were oversampled. 19% of invited participants were included and represented approximately 11% of the population of NSW aged 45 years and over. Between 2020 and 2022 a sub-sample of 32,115 participants from the original study were recruited to participate in the COVID Insights study [21]. This was a series of five supplementary surveys that provided information about how participants experienced the COVID-19 pandemic. Consent and participation were completed via an online platform and took approximately 10 minutes per survey. We primarily analysed data from survey 3 (collected between June and August 2021). We obtained one variable (social support) from survey 1 (collected between November and December 2020) as it was not measured in survey 3. Since we were interested in analysing social support derived from survey 1, we excluded participants who did not respond to both surveys. Given that we were interested in the vaccination intentions of older Australians, we also excluded participants aged < 60 based on the World Health Organization’s definition of ‘older persons’ [22].

### Measures

From survey 3, we analysed measures relevant to potentially traumatic life events, psychological wellbeing, and vaccination intentions. The experience of potentially traumatic events was measured with the item; ‘In the last 12 months, have you suffered a serious illness, injury or assault’ (yes/no). A shortened version of the Kessler Psychological Distress Scale (K6) [23] was used as a measure of psychological distress. It consists of 6 items and is intended for use as a rapid assessment of risk for serious mental illness. Respondents are asked whether they have had six different experiences in the past 30 days and indicate frequency on a 5-point Likert scale ranging from “all of the time” to “none of the time”. Examples of items include “during the past 4 weeks, about how often did you feel nervous?” and “during the past 4 weeks, about how often did you feel worthless?”. The total K6 score was calculated by summing the five possible points for each of the six items. Intention to receive a COVID-19 vaccine was measured with the question “Do you intend to get a COVID-19 vaccine?”. Response options were “yes”, “no” and “don’t know”. The response options “no” and “don’t know” were combined to form the dichotomous measure of intention of “no/don’t know” and “yes”.

A measure of social support was derived from survey 1 and obtained using the abbreviated Duke Social Support Index (DSSI) [24]. This tool consists of 10 items with two subscales: (1) four items measure social interaction, for example, “how many times in the past week did you spend time with someone who does not live with you, that is, you went to see them or they came to visit you or you went out together?” (the 8-point response scale ranged from “0” to “7 or more times”) (2) six items measure satisfaction with social support for example, “Does it seem that your family and friends understand you?” (response options were “hardly ever”, “some of the time” or “most of the time”). One of these six items asked; “How many people outside your home, but within one hour of travel, do you feel you can depend on or feel close to?”, and thus had different response options (“none”, “1–2 people, “more than 2 people”). The DSSI was scored by summing scores across both subscales, with higher scores indicating more social support.

### Analysis

We used SPSS version 27 to analyse the data. To assess whether potentially traumatic events, psychological distress and social support could predict intention to receive a COVID-19 vaccine, we first conducted bivariate analyses; two independent samples t-tests to assess the relationship between intention and (i) social support and (ii) psychological distress; and a chi-square test to assess the relationship between intention and exposure to a potentially traumatic event in the last 12 months. Any associations with a significance level of *p* <.2 in a bivariate analysis (measures of social support, psychological distress, and personally experiencing a serious illness, injury or assault in the last 12 months), were included in a logistic regression model predicting intention.

To assess how levels of social support and psychological distress are associated with age, we conducted bivariate regression using age to predict DSSI and K6 scores. We then conducted a binary logistic regression to assess if belonging to a particular age group (60–69, 70–79, 80–89 and 90–99) could predict the experience of a potentially traumatic life event in the last 12 months. We used an alpha level of 0.05 for all statistical tests and 95% confidence intervals for all regression analyses.

## Results

A total of 27,012 participants responded to survey 3. We excluded those who were < 60 years of age (*n* = 1069) and those who were recruited between the first and third survey and therefore did not complete survey 1 (*n* = 12,972). There were 3135 respondents who were either lost to follow-up between survey 1 and survey 3 or had missing data for measures on one of the surveys. These were excluded, leaving a final sample of 12,971. Participants resided in New South Wales (NSW), Australia’s most populous state. Most respondents were from major cities (57.6%, *n* = 7,471), followed by regional areas (41.7%, *n* = 5,413) and remote areas (0.5%, *n* = 64). Respondents’ ages ranged from 60 to 98 years (M = 69.9, SD = 6.6), 56% (*n* = 7,235) identified as female, and 44%, (*n* = 5,736) as male. Most respondents (93.6%, *n* = 12,135), intended to receive a COVID-19 vaccine. The remaining 6.4% (*n* = 836) either did not intend −or did not know if they intended− to receive a COVID-19 vaccine. A total of 10,102 respondents had received at least one dose of a COVID-19 vaccine. One-thousand three hundred and forty-one (10.3%) respondents had personally experienced a serious illness, injury or assault in the last 12 months. While social support was negatively skewed in our sample, with DSSI scores ranging from 10 to 30 (M = 24.8, SD = 3.3), levels of psychological distress were positively skewed, with K6 scores raging from 6 to 30 (M = 8.8, SD = 3.1). Although the data exhibited skew, it was not severe enough to violate test assumptions, especially given the large sample size. All other assumptions for statistical tests were met.

### Associations with intention to receive a COVID-19 vaccine

An independent samples t-test indicated a statistically significant difference in social support between those who reported intending to receive a COVID-19 vaccine and those who did not, t(913.7) = 7.7, *p* <.001. Mean DSSI score (social support) was higher in those who intended to receive a vaccine (M = 24.9, SD = 3.3) compared to those who did not intend to, or were not sure if they intended to, receive a vaccine (M = 23.8, SD = 4.01); the difference in means of 1.1(95% CI: 0.81–1.37) representing a medium effect (r^2^ = 0.3). A second independent samples t-test indicated a statistically significant difference in psychological distress between those who intended to receive a COVID-19 vaccine and those who did not, t(912.2) = 4.87, *p* <.001. Those who had higher levels of psychological distress had lower vaccination intentions as mean K6 score was lower in those who intended to receive a vaccine (M = 8.47, SD = 3.12) compared to those who did not intend or were not sure if they intended to receive a vaccine (M = 9.13, SD = 3.85); the difference in means of 0.66 (95% CI: 0.4–0.93) representing a small effect (r^2^ = 0.19). These results are summarised in Table 1.

**Table 1:** Bivariate associations between psychosocial variables and intention to receive a COVID-19 vaccine

Variable	Intention to receive a COVID-19 vaccine Yes No/not sure				***t***	***p***	***r*** ^2^
	*M*	*SD*	*M*	*SD*			
Social support (DSSI score)	24.9	3.3	23.4	4.01	7.7	< 0.001	0.3
Psychological distress (K6 score)	8.47	3.12	9.13	3.85	4.87	< 0.001	0.19

A chi-square test was performed to examine the relationship between exposure to a potentially traumatic event in the last 12 months and intention to receive a COVID-19 vaccine. The relationship was significant χ^2^(1, *N* = 12,971) = 9.02, *p* =.003. People who were willing to receive a COVID-19 vaccine were less likely to experience a potentially traumatic event in the last 12 months. Frequencies of the variables in the chi-square test are shown in Table 2. Given significant associations between vaccination intention and (i) social support; (ii) psychological distress; and (iii) personally experiencing a serious illness, injury or assault in the last 12 months, these variables were retained for inclusion in the logistic regression analysis predicting vaccination intention. In the final model of the logistic regression shown in Table 2, higher intention to receive a COVID-19 vaccine was associated with increased social support (DSSI score) and lower intention was associated with personally experiencing a serious illness, injury or assault in the last 12 months compared to not having this experience. Level of psychological distress (K6 score) did not contribute to intention to receive a COVID-19 vaccine in the final model.

**Table 2:** Correlates of COVID-19 vaccine intention

	Participants intending to receive a COVID-19 vaccine/ total participants in response category n(%)		Unadjusted Odds Ratio (95% CI)	***P***	Adjusted Odds Ratio (95% CI)	***P***
Social support		-	1.09 (1.07–1.11)	< 0.001	1.08 (1.06–1.11)	< 0.001
Psychological distress		-	0.99 (0.97–1.01)	0.31	0.99 (0.97–1.02)	0.55
Experienced potentially traumatic event in the last 12 months	Yes	1229/1341 (91.65)	0.78 (0.64–0.97)	0.02	0.79 (0.64–0.98)	0.03
	No	10,906/11,630 (93.78)	1		1	

### Associations with age

Given that psychosocial variables were found to be associated with the vaccination intention of older Australians, we sought to assess if and how these may also be associated with age. This was done to better understand of the needs of different age groups within the cohort of older Australians. Simple linear regression was first used to predict levels of social support and psychological distress using age. Age explained a significant amount of the variance in social support, F(1,12969) = 55.34, *p* <.001, R^2^ = 0.004. The regression coefficient (B = 0.07, 95% CI[0.024,0.042]) indicated that an increase in social support (DSSI score), would correspond to an increase in age. Age also explained a significant amount of the variance in psychological distress, F(1,12969) = 119.97, *p* <.001, R^2^ = 0.009. The regression coefficient (B = 0.05, 95% CI[0.04–0.05]) indicated that an increase in psychological distress (K6 score), would correspond to a decrease in age. A binary logistic regression was conducted to see if age groupings (60–69, 70–79, 80–89 and 90–99) could predict the experience of a traumatic event in the last 12 months. The model is represented in Table 3. The odds of experiencing a traumatic event increased significantly with each increasing age group compared to the youngest age group (60–69).

**Table 3:** Logistic regression model for age group and intention to receive a COVID-19 vaccine

Age group (years)	Participants who experienced a potentially traumatic event in the last 12 months / total participants in age group n (%)		Odds Ratio (95% CI)		***P***
60–69	605/6556 (9.23)	1		-	
70–79	578/5323 (10.86)	1.2 (1.06–1.35)		0.003	
80–89	144/1010 (14.26)	1.64 (1.35–1.2)		< 0.001	
90–99	14/82 (17.07)	2.02 (1.13–3.62)		0.02	

## Discussion

We found that increased intention to receive a COVID-19 vaccine was associated with greater social support among older NSW residents, while decreased intention was associated with the experience of a potentially traumatic event within the last twelve months. Social support and the experience of potentially traumatic events increased significantly with age while psychological distress decreased. While increased psychological distress was independently related to lower vaccination intention, it did not contribute to intention when it was considered alongside social support and the experience of a traumatic event. This might reflect how trauma and psychological distress were measured. Although both were independently related to decreased intention, the measure of a potentially traumatic experience may have elicited memories of a specific event, while the measure of psychological distress was more general and not necessarily related to a single experience. Given that a traumatic event may condition an individual to view an innocuous stimulus —in this case vaccination— as threatening [25, 26], our measure of trauma may have been more influential in vaccination decision-making when considered alongside a generalised measure of psychological distress.

While studies show that increased age is associated with increased vaccination intention [4, 27], our findings suggest additional contributors to this trend. Despite having experienced more traumatic events in the last twelve months, older people were still less likely to experience psychological distress with increased age. This may be explained by our finding that social support increases with age since psychological distress is associated a lack of social support in older population groups [28, 29]. Accordingly, older Australians who are at the younger end of the age bracket have higher levels of psychological distress and less social support than those in older age groups and these factors have been associated with decreased vaccination intention. Interestingly, vaccine uptake data from September of 2021 reflect this finding, with fewer first doses taken by those aged 60–69 (78.5%) compared to older age groups; 70–79 (87.6%), 80–89 (87.7%) and aged over 90 (86.3%). While older age groups may have been able to access the vaccine more easily through residential aged care, it might also suggest that different age groups within the older age spectrum can have different needs. Understanding the needs of specific age groups, particularly those aged 60–69, may be helpful when tailoring interventions for older Australians.

We found that social support contributes to increased vaccination intention. This mirrors findings from studies of younger adult populations where online social support increases COVID-19 vaccination intention [30], and loneliness decreases the likelihood of engaging in preventative behaviours for COVID-19 [31]. Our measure of social support was collected approximately 7 months prior to all other measures in our study which gives more ground for a causal relationship with vaccination intention. It might be the case that perceived social support remained stable over that period: 38% of Australians aged over 65 require assistance with daily tasks and the prevalence of chronic conditions and mobility issues increases with age [32]. Those who have support from carers might also have assistance planning to receive a vaccine which might explain our findings. Conversely, a qualitative study found that older people who lack social support are concerned that they might have no help should they experience an adverse event following immunisation, and that this contributes to hesitancy [33]. Interventions aimed at vaccine hesitant older Australians might benefit from targeting other adults who are able to offer their support by helping to facilitate vaccination and provide assistance following vaccination if needed.

Previous studies have shown that traumatic events are associated with decreased vaccination intentions in adult populations and for child vaccination decisions made by parents who have experienced trauma [10, 11, 34]. The present study furthers this knowledge by contributing insights about vaccination decisions made by older adult populations who have experienced a traumatic event in the last twelve months. Psychological trauma is seemingly less prevalent in older compared to younger adults [35] however, cohort effects might influence this reporting as most older adults grew up during a time when the effects of trauma on psychological wellbeing were not commonly understood [36]. Unlike previous studies, this survey used a single simple question to screen for trauma exposure. Although this may miss the nuance of the traumatic event, the fact that we found an association with vaccination intention suggests that one approach to addressing vaccine hesitancy in older Australians is to consider potential exposure to trauma in the therapeutic consultation. However, further qualitative research is first needed to understand the reasons for this relationship. Moreover, given our finding that social support is related to vaccination intention, further research might explore the mediating relationship of social support in the relationship between traumatic experiences and vaccination intention.

Our study has some limitations. Some respondents were excluded from the final analysis which has the potential to introduce bias. While the majority of these were newly recruited between surveys and do not contribute to attrition bias, we do not know how variables differed in those lost-to follow-up, or excluded. Many respondents had already received at least one dose of a COVID-19 vaccine. Given that the COVID-19 booster vaccination program did not commence until the end of 2021, participant intention reflected at least one prospective COVID-19 vaccine. Intention to receive a vaccine may have differed depending on how many the respondent had received in the past, or whether they had ever been diagnosed with COVID-19. These factors were not explored in this study. Also, data were only collected from residents of one Australian state so generalisation of our findings to the broader Australian population should be done with care. The same is true for cultural sub-groups whose unique experiences may influence their vaccination decisions [37, 38]. Future studies could compare older Australians within different states and cultural groups to facilitate comparisons and the development of targeted vaccination interventions. Finally, our question measuring potentially traumatic events is general and does not give us specific detail about the nature of the event itself. Qualitative explorations, or a more pointed line of questioning using validated psychometric measures of trauma, may give insight into how the particulars of a traumatic experience contribute to the vaccination intentions of older Australians.

## Conclusions

Increased vaccination intention of older Australians residing in NSW is associated with increased social support, and decreased intention with the experience of a potentially traumatic event. Older age is associated with increases in social support, the experience of potentially traumatic events, and a decrease in psychological distress. Although older Australians have been targeted by vaccination programmes due to their increased disease-associated risks, there may be more factors that play a role in increased vaccine acceptance with age. Older Australians on the younger end of the age spectrum may have specific needs to address their hesitancy that may be overlooked. Considering potential exposure to trauma when addressing hesitancy in clinical settings and targeting the social support networks of older Australians may be helpful for vaccination interventions.

## Data Availability

The data that support the findings of this study are available from The Sax Institute, but restrictions apply to the availability of these data, which were used under license for the current study, and so are not publicly available. Any queries may be addressed to the corresponding author.
